# A novel degradable PCL/PLLA strapping band for internal fixation of fracture

**DOI:** 10.1007/s10856-023-06759-7

**Published:** 2023-11-08

**Authors:** Baoyan Jin, Chongjing Zhang, Zeyuan Zhong, Zichen Liu, Zhenhua Zhang, Dejian Li, Min Zhu, Baoqing Yu

**Affiliations:** 1 School of Materials and Chemistry, University of Shanghai for Science and Technology, Shanghai, China; 2 Department of Orthopedics, Shanghai Pudong New Area People’s Hospital, Shanghai, China; 3 Department of Orthopedics, Shanghai Pudong Hospital, Fudan University Pudong Medical Center, Shanghai, China

## Abstract

Early fracture fixation is the critical factor in fracture healing. Common internal fracture implants are made of metallic materials, which often affects the imaging quality of CT and MRI. Most patients will choose secondary surgery to remove the internal fixation implants, which causes secondary damage to them. The development of new degradable internal fracture implants has attracted more and more attention from orthopedic surgeons and researchers. Based on these problems, we improved the various properties of medical grade polycaprolactone (PCL) by adding poly(L-lactide) (PLLA). We produced PCL/PLLA strapping bands with different mass ratios by injection molding. We compared the mechanical properties, degradation properties, cell biocompatibility, bone marrow mesenchymal stem cells (BMSCs) adhesion, proliferation, osteogenic differentiation and fracture fixation effect of these strapping bands. The results showed that the tensile strength and yield force of the strapping bands increased with the increase of the content of PLLA. The addition of PLLA could significantly improve the mechanical strength in the early stage and accelerate the degradation rate of the strapping band. PCL/PLLA (80/20) strapping band had no significant cytotoxicity toward rBMSCs and could promote osteogenic differentiation of rBMSCs. The strapping band could ensure femoral fracture healing of beagles in 3 months and didn’t cause damage to the surrounding tissues and main organs. This study will provide some new insights into the biodegradable products of PCL/PLLA blends for internal fixation of fracture.

**Graphical Abstract:**

**Figure:**
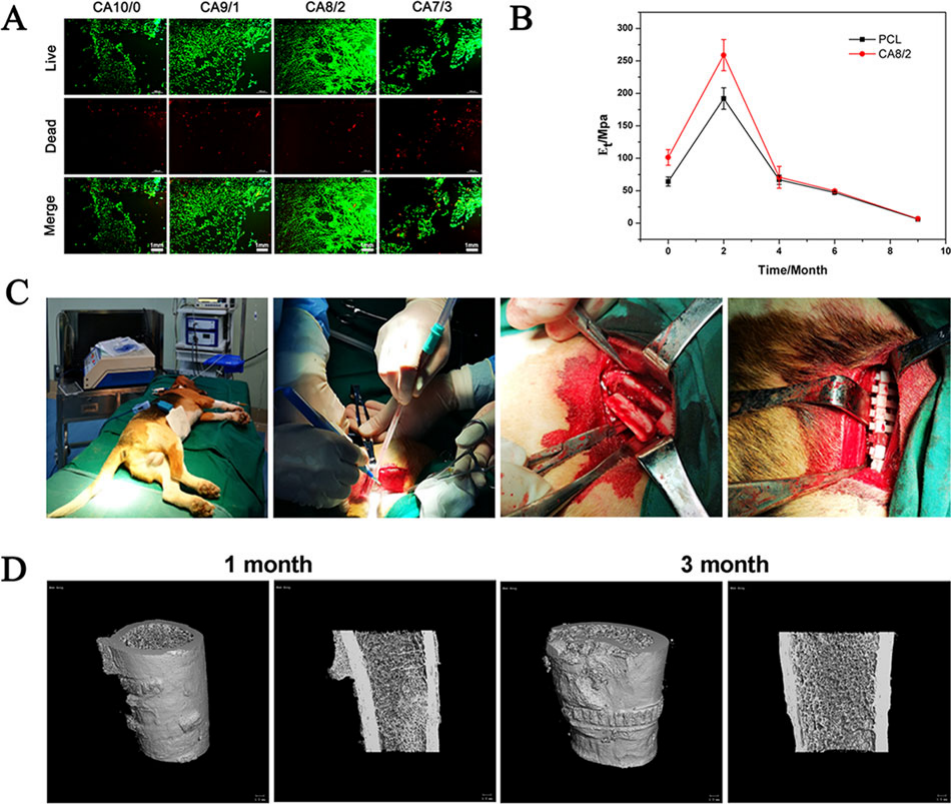
We produced novel degradable PCL/PLLA strapping bands with different mass ratios by injection molding. We tested the biological safety of the prepared internal fixation strapping bands for fracture, such as cell experiment in vitro and animal experiment, and studied the degradation behavior in vitro. The strapping bands could ensure femoral fracture healing of beagles. This study will provide some new insights into the biodegradable products of PCL/PLLA blends for internal fixation of fracture. **A** Immunofluorescence staining of rBMSCs (live cells: green; dead cells: red). **B** Young’s modulus change curve during strapping bands degradation. **C** The implantation process of strapping bands. **D** Micro-CT images of the beagle’s fracture recovery after the operation.

## Introduction

Fractures are common in orthopedics and their repair often include reduction, fixation, and functional exercise [1]. Fixation is a crucial part of the rehabilitation process, which determines the ultimate recovery of function. Fixation methods can be divided into external fixation and internal fixation. External fixation has minimal damage to soft tissue and the position of external fixation can be adjusted according to the rehabilitation needs [2]. However, external fixation leads to a higher incidence of malunion and nonunion of bone and restricts limb movement in the early stage of healing process. Internal fixation can fix the bone fragments in the anatomical positions. Anatomical reduction can make the fracture grow stably in a good position without affecting the activity if the mechanical properties of the internal fixation device are sufficient [2, 3].

There are over 3 million fractures in the United States each year, over 30% of which require internal mechanical fixation devices to aid in the healing process [4]. Alloys are the most commonly used materials for the preparation of internal fixation devices. However, alloys internal fixation devices have many disadvantages, such as stress sheltering, metal ion leaching, artifacts in imaging examination, and the need for secondary surgery to remove the devices after healing [5, 6]. Polymer and its composite materials are hotspots in the field of biomedical materials in recent years. In the 1960s, many countries started the research on biodegradable polymer materials, aiming to find internal fixation materials that can not only meet the mechanical properties of fracture internal fixation, but also can be gradually degraded, absorbed or excreted in the body [7]. The biodegradable polymer materials that used for internal fixation of fracture should have good biocompatibility, no antigenicity, no rejection, no carcinogenicity, no teratogenicity and other general characteristics. They should have appropriate degradation speed and matching mechanical strength with the healing of fractures and be easy to mold, disinfect, and preserve.

As a biodegradable polymer, polycaprolactone (PCL) has significant advantages, such as good biosafety, excellent biodegradability, good toughness, low melting temperature, variable viscosity and strong versatility and PCL is very suitable for melt processing, such as injection molding, melt extrusion, 3D printing and electrostatic spinning technology [8, 9]. However, the application of PCL in internal fixation of fracture is limited by its long degradation cycle, general mechanical properties and high cost [10]. Therefore, PCL is usually compounded with other biodegradable materials to improve its performance. In recent years, the mechanics and degradation properties of PCL-based biodegradable polymer composites have been studied extensively.

The addition of gelatin can significantly improve the tensile strength of PCL, up to 3 times, reduce the water contact angle and improve the hydrophilicity, compared with the pure PCL. The higher the gelatin content, the faster the degradation rate in vitro [11–13]. Liu, et al. [14] prepared PCL/PGS scaffolds with a weight ratio of 1: 1 by salting out method, and its elastic modulus was nearly 10 times higher than that of pure PCL. The mass loss of PCL/PGS scaffolds at each node was higher than that of pure PCL after 30 days of in vitro degradation. The elastic modulus of PCL/PGS scaffolds during degradation was higher than PCL group. Hedayati, et al. [15] prepared PCL/PGA scaffolds by 3D printing, and their tensile strength and modulus were increased by 374% and 775%, respectively. After 10 weeks of degradation in Roswell Park Memorial Institute (RPMI) 1640, the mass loss of PCL/PGA scaffolds was 20 times than the pure PCL. The elastic modulus of PCL/PGA scaffolds decreased by 96% and the pure PCL decreased by 48%. Ren, et al. [16] prepared the PCL/PEG composite mesh by melt electrowriting (MEW), and the yield strength and Young’s modulus of the PCL/PEG composite mesh were increased by 276% and 615%, respectively, compared with pure PCL. After accelerated degradation in NaOH solution, PCL/PEG composite mesh could not be retrieved after 10 h, while the pure PCL mesh can still be retrieved after 3 days.

However, previous research only focused on the basic material properties and short-term degradation, lacking data on long-term degradation, biosafety performance, and animal experiments. Polylactic acid (PLA) is a biodegradable polymer with fast degradation rate, good mechanical properties and processing performance [17, 18]. These characteristics are complementary to PCL. In addition, PCL and PLA have been approved by the Food and Drug Administration (FDA), and PLA products have been widely used in clinical. In this paper, Poly(L-lactide) (PLLA) and PCL were blended to prepare PCL/PLLA strapping bands with dentate grooves for internal fixation of fracture by injection molding, and the mechanical properties, biosafety in vitro and vivo, and long-term degradation of PCL/PLLA strapping bands were studied. This will provide a certain basis for the transformation of achievements in the future.

## Methods

### Materials

Poly(-ε-caprolactone) (PCL) (RESOMER^®^ C 212, inherent viscosity is 1.13–1.38 dL/g), Poly(L-lactide) (PLLA) (RESOMER^®^ L 206 S, inherent viscosity is 0.8–1.32 dL/g), Poly(L-lactide-co-ε-caprolactone) (PLCL) (RESOMER^®^ LC 703 S, inherent viscosity is 1.3–1.8 dL/g) were purchased from the Evonik Operations GmbH, Darmstadt, Germany.

### Preparation of PCL/PLLA strapping bands

PCL was dried at 45 °C and −0.09 Mpa for 4 h in a vacuum drying oven, PLLA was dried at 110 °C and −0.09 Mpa for 2 h in a vacuum drying oven. According to the mass ratio of 100: 0, 90: 10, 80: 20, 70: 30, the strapping bands were recorded as CA10/0, CA9/1, CA8/2 and CA7/3, respectively, mechanical blending is carried out in the mixer, and the PCL/PLLA strapping bands is obtained by injection molding of the evenly blended mixture by injection molding machine (ClassiX 35-55CX, KraussMaffei, Munich, Germany). The barrel temperature is 170–185 to 185–175 °C, the mold pressure is 320 kN, and the injection stroke is 9.5 mm. The injection speed was 150 mm/s, the pressure holding time was 7.0 s, the injection pressure was 2000 bar, and the cooling time was 18 s.

### Characterization of PCL/PLLA strapping bands

The morphological analysis of the strapping bands was characterized by the scanning electron microscopy (SEM, GeminiSEM 300, Zeiss, Germany). After the strapping bands was brittle-broken by liquid nitrogen, the section was sprayed with vacuum gold and transferred to the scanning electron microscope for testing. The acceleration voltage was 15 KV.

The glass transition temperature and melting temperature analyses of the strapping bands were characterized using the differential scanning calorimeter (DSC, DSC 200F3, Netzsch, Germany). The test temperature range was −80~200 °C. In N_2_ atmosphere, the temperature was raised from room temperature to 200 °C at a rate of 10 °C/min, and the heat history was eliminated at a constant temperature of 3 min. Then the temperature was lowered to −80 °C for 3 min, and finally the temperature was raised to 200 °C for 3 min.

The molecular structure analysis of the strapping bands was characterized using Fourier transform infrared spectrometer (FTIR, Nicolet iS 50, Thermo Fisher, USA). We can determine the chemical structure of the material according to the position and shape of the absorption peak. The test wavelength ranged from 400 μm to 4000 μm.

Tensile properties were tested using the electronic universal testing machine (UTM17339, SUNS, China) in water bath constant temperature circulation system at 37 °C. The strapping bands ware subjected by unilateral axial tension and lock tension respectively, and its tensile strength and yield force were analyzed.

### Biological safety testing

#### Isolation and culture of rBMSCs

Bone marrow mesenchymal stem cells (rBMSCs) were extracted from Sprague Dawley (SD) rats for cell experiments [19–21]. Four 2-week-old SD rats were sacrificed and immersed in alcohol for 10 min. Femur and tibia were taken and transferred to a petri dish filled with PBS solution. After stripping muscle and soft tissue, the femur and tibia were placed in a 1.5 mL conical centrifuge tube immediately. After centrifugation at 12,000 RPM at 4 °C for 1 min, the femur and tibia in the centrifuge tube were removed, the centrifuged precipitate was transferred to the centrifuge tube after re-suspension with 1 × PBS, and 5 times the volume of erythrocyte lysate was added. After re-suspension, and the centrifuge was repeated two times (1000 RPM/min, 5 min). After the supernatant was removed, PBS was added and centrifuged for the third time (1000 RPM/min, 5 min) after suspension. Finally, the cells were evenly placed into a culture dish with a diameter of 10 cm with a rubber dropper and cultured in the cell culture box with a controlled atmosphere (5% CO_2_/95% air, 90% hygrometry and 37 °C). After 24 h, the culture medium was replaced entirely, and the cells were observed under a microscope to differentiate from punctured cells to long spindle cells. The cells were then transferred into the cell culture box, and the cell culture medium was replaced every 3 days after that. When the cells were cultured to 80%~90% confluence, the primary rBMSCs were digested with trypsin and then passed on to the third generation, which could be used for cell experiments of strapping bands.

#### Adhesion of rBMSCs to PCL/PLLA strapping bands

The ethylene oxide-sterilized strapping bands were put into 24-well plates, with 3 in each group, and rBMSCs were inoculated on the upper surface of the bands at the rate of 5 × 10^4^ cells/well.

Take the strapping bands and place them in the center of the wells of the culture plate in sequence, add the calculated dose of cell stock solution to each well, and place them in an incubator for 2 h. The complete medium preheated with 37°C warm water was slowly added into the well according to 1 mL per well, the cells were observed under the microscope, put into the incubator, and the cell culture medium was changed every 3 days.

After 7 days culture, we used Calcein-AM/PI Live/Dead cell double staining Kit (Solarbio, China) to determine the ratio of live and dead cells, in which live cells appear green at 490 nm fluorescence and dead cells appear red at 545 nm fluorescence [22].

#### Proliferation of rBMSCs on PCL/PLLA strapping bands

The strapping bands were placed in the center of each well on the 24-well plate, and 5 × 10^4^ cells/well were inoculated. Cell viability was detected by Cell Counting Kit-8 (CCK-8) (MCE, USA) according to the kit manufacturer’s protocol.

#### Alkaline phosphatase activity of rBMSCs on PCL/PLLA bands was detected

RBMSCs were inoculated with 5 × 10^4^ cells/well in 24-well plates. After the cells were fused to 80%, osteogenic induction solution (complete medium as solvent, β -sodium glycerophosphate concentration of 10 mM/L, ascorbic acid 50 μmol/L) was added. Dexamethasone (0.1 μmol/L) was used for osteogenic differentiation induction instead of complete medium [23]. After 3 and 7 days of induction, alkaline phosphatase activity was detected using the ALP kit (Thermo Fisher, USA) according to the kit manufacturer’s protocol.

#### Expression of osteocalcin recombinant protein on PCL/PLLA strapping bands of rBMSCs

RBMSCs were inoculated with 5 × 10^4^ cells/well in 24-well plates. After the cells were fused to 80%, osteogenic differentiation was induced with the osteogenic induction solution instead of the complete medium. Osteocalcin recombinant protein was detected by double-antibody sandwich enzyme-linked immunosorbent assay (ELISA) kit (MeiMian, China) according to the kit manufacturer’s protocol.

### In vitro degradation test of PCL/PLLA strapping bands

In vitro degradation performance test: PCL and PLLA were easily degraded by hydrolysis [24–27]. Phosphate buffered saline (PBS) was used to simulate in vivo degradation conditions [28], the pH value was 7.40 ± 0.01, and the temperature was 37 °C. The strapping bands were divided into two groups, pure PCL strapping bands as the control group and PCL/PLLA strapping bands as the experimental group. The strapping bands were locked into a loop and put into a 100 mL reagent bottle, and were submerged by PBS. The bottle was tightened and placed in a 37 °C constant temperature biochemical culture box, and PBS was replaced once a month. At 0, 2, 4, 6, 9, 14, and 18 months, the pH value of PBS, molecular weight and mechanical properties of the strapping bands were tested, and the morphology of the strapping bands was analyzed at each stage.

### In vivo safety assessment of PCL/PLLA strapping bands

Ten adult male beagles with the body weight of 10–15 kg were purchased from Shanghai Jiagan Biotechnology Co., Ltd (Shanghai, China). The ten beagles were randomly divided into two groups (5 dogs in each group). The control group was treated with traditional plaster external fixation, and the experimental group was treated with PCL/PLLA bands internal fixation.

Inject the anesthetic sutai to the beagle, and subcutaneously inject atropine sulfate at a dose of 0.1 mg/kg in the first 15 min, the anesthetic dose of 5 mg/kg, intravenously. Expose the mid-end of the femur, use a high-speed electric abrasive sheet to cut a rectangular bone block of about 1 × 3 cm. After the bone block is completely detached, put the bone block back to its original position and fix it with a bandage, and then suture and apply erythromycin ointment in sequence. 200,000 U/day/dog of penicillin and 10 mg/kg/day of gentamicin were injected for a total of 5 days.

After the rehabilitation period, blood samples were collected to analyze inflammatory factors and trace elements in the body. HE staining and Masson staining were performed on some connective tissues around the band to observe whether there were tissue reactions and fibrous envelope formation at the fracture site, and whether the band fell off, shifted or fractured. All animal experiments in this study were carried out in Pudong Hospital of Fudan University in strict accordance with the relevant regulations of the Animal Ethics Committee of Pudong Hospital of Fudan University.

### Statistical analysis

All experiments were repeated 3 times, and the results are presented as the $\bar{x}$ ± SD for data that were normally distributed. In this study, GraphPad Prism (version 8, GraphPad Software, San Diego, USA) was used for statistical analysis. Comparisons between two groups were performed by Student’s *t*-test for normally distributed variables or Mann–Whitney *U* test for nonnormally distributed variables. One-way ANOVA with Tukey’s post-hoc test was used to determine whether the differences among several groups were statistically significant. Values of *P* < 0.05 were considered to indicate statistically significant differences.

## Results and discussion

### Characterization of the PCL/PLLA strapping bands

**Fig. 1:**
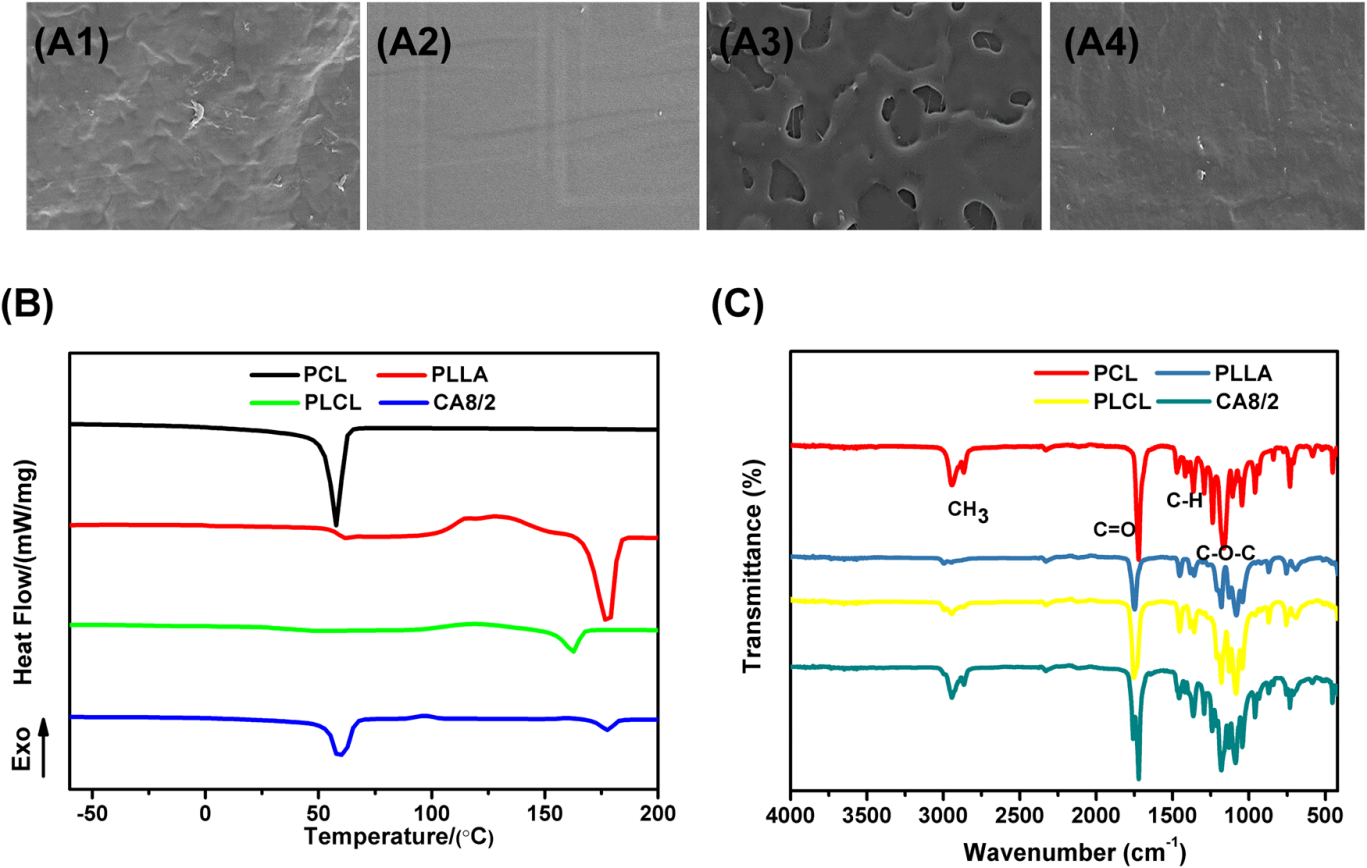
**A1**–**A4** are SEM images of PCL, PLLA, PLCL, and CA8/2 strapping bands (50 μm), **B** DSC curves of PCL, PLLA, PLCL and CA8/2 strapping bands, and **C** Fourier infrared spectrum of PCL, PLLA, PLCL and CA8/2 strapping bands

As shown in Fig. 1A1–A4, in order to verify whether the PCL/PLLA strapping bands obtained by injection molding were uniform, PCL, PLLA, and PLCL strapping bands were taken as the control group, and CA8/2 strapping bands showed SEM morphology as the experimental group. The sections of PCL, PLLA, and PLCL are uniform, only quenched, without incompatible interface; CA8/2 is flat, without obvious incompatible interface in the image, indicating that the uniformity between PCL and PLLA is good.

As shown in Fig. 1B, the secondary heating DSC curves of PCL, PLLA, PLCL, and CA8/2 were obtained by differential scanning calorimetry. It shows that the melting peak of the PCL occurs at 56 °C, or the melting temperature is 56 °C. Similarly, PLLA has a melting temperature of 180 °C and a glass transition temperature of about 60 °C, with a broad peak of cold crystallization between *T*_g_ and *T*_m_. PLCL is a random copolymer of PCL and PLLA, with only one characteristic melting peak, where the corresponding *T*_m_ is around 160 °C. Two characteristic melting peaks appeared in CA8/2, corresponding to the peaks at around 57 °C and 178 °C, respectively, which agree with those of PCL and PLLA. According to the melting temperature, we can determine whether the system is a random copolymer or a blend [29]. The PLCL has only one melting peak, while CA8/2 has its respective melting peak in each component region, which proves the thermodynamic incompatibility of PCL and PLLA.

Figure 1C shows the FTIR spectrum of PCL, PLLA, PLCL and CA8/2. For PCL, the characteristic peaks of the hydroxyl group occur at 3440–3500 cm^−1^. The peak intensities of end groups are always weak due to their low molar content in the corresponding polymer chain. Two different peaks, ranging from 2850 to 2980 cm^−1^, correspond to the asymmetric and symmetric stretching modes of the -CH_3_, respectively. Base expansion vibration peaks occur at 1715~1755 cm^−1^, while C-O-C expansion vibration peaks occur at 1050–1200 cm^−1^. Peaks at 1430 to 1480 cm^−1^ represent the -CH deformation, while the peaks at 733 to 756 cm^−1^ represent the skeleton vibration of the -CH_2_. According to the FTIR analysis, CA8/2 corresponding to the peak of C = O at 1720 cm^−1^ is shown as two peaks, indicating that the adhesive motion between PCL and PLLA is different, but the blends did not change the characteristic functional groups.

**Fig. 2:**
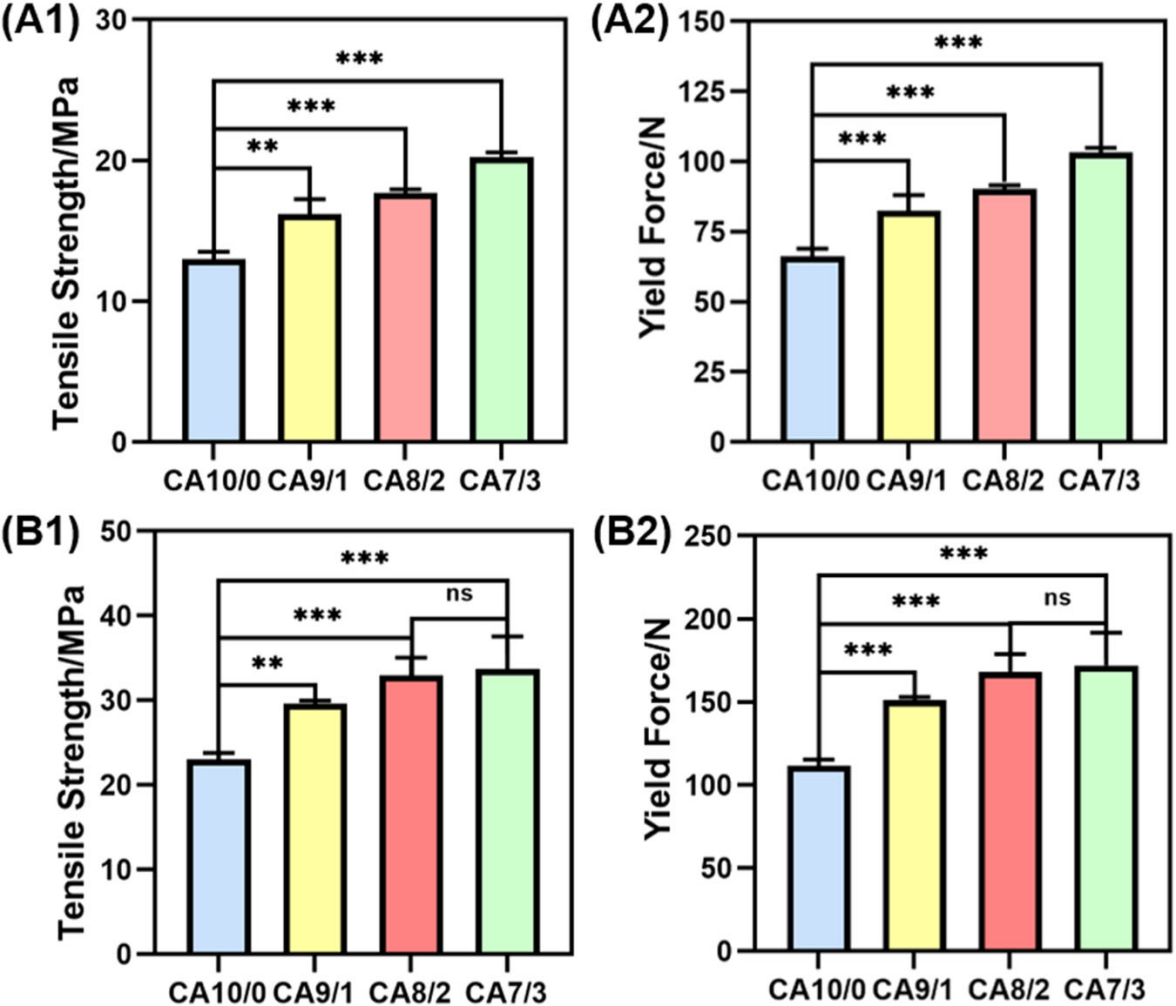
PCL/PLLA tensile test for different mass ratios: **A1** tensile strength of unilateral axial stretch, **A2** yield force of unilateral axial stretch, **B1** tensile strength of lock stretch, **B2** yield force of lock stretch (*n* = 5, *p < 0.05, **p < 0.01, ***p < 0.001)

The results of PCL/PLLA tensile test with different mass ratios are shown in Fig. 2. It is observed from Fig. 2 that the yield force and tensile strength increase with unilateral axial stretching and PLLA content, indicating that PLLA can significantly enhance the mechanical strength of PCL. In the unilateral axial tensile test, the tensile strength of CA9/1, CA8/2, and CA7/3 was increased by approximately 24.5%, 35.9%, and 55.5% over CA10/0, respectively. The yield force has been increased from 66.3 N of CA10/0 to 103.2 N of CA7/3, increased by about 55.7%. During the latch stretch test, the tensile strength of CA9/1, CA8/2, and CA7/3 is about 27.8%, 43.3%, and 46.6% greater than that of CA10/0, respectively. The yield force from 111.8 N of CA10/0 to 172.1 N of CA7/3, the increase is about 54.0%. However, there is no significant difference in the tensile strength and yield force between CA8/2 and CA7/3. In addition, the tensile strength and yield force of the lock stretching are greater than the unilateral axial stretching.

### Biological safety testing

**Fig. 3:**
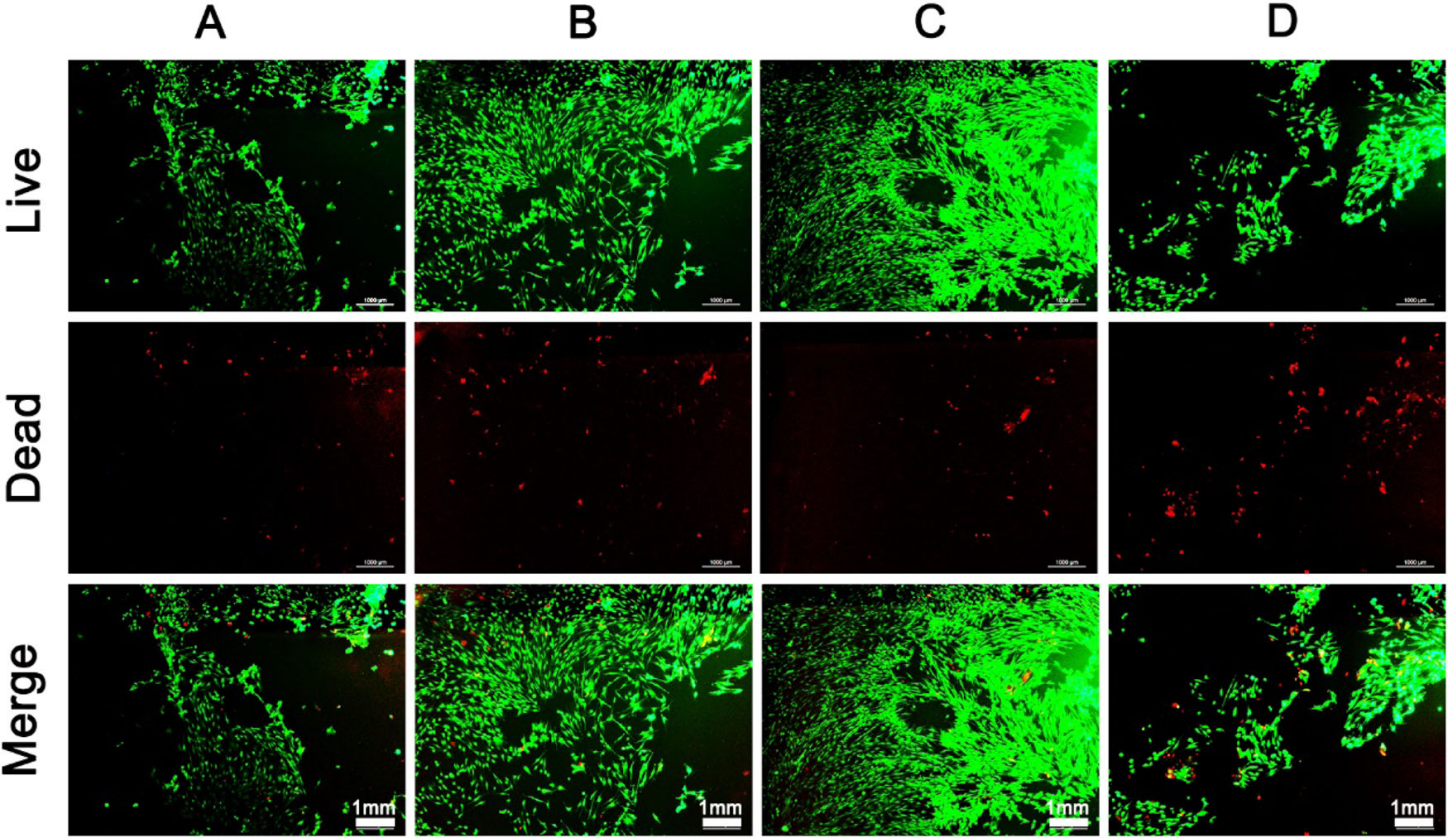
Immunofluorescence staining of rBMSCs (live cells: green; dead cells: red): **A** CA10/0, **B** CA9/1, **C** CA8/2, **D** CA7/3 (1 mm)

To evaluate the osteogenic capacity of PCL/PLLA strapping bands, rBMSCs were seeded on PCL/PLLA strapping bands with different mass ratios, and rBMSCs adhesion status was observed under the immunofluorescence microscope on the 7th day, using the surface with dentate grooves of the strapping bands as the image to obtain fluorescent staining images of live/dead cells as shown in Fig. 3. Living cells were green and dead cells were red. Compared with CA10/0 strapping bands, the area of living cells in CA8/2 strapping increased significantly. There were the most densely distributed, while the area of corresponding dead cells did not increase. However, the area of live cells in CA7/3 strapping bands decreased significantly and the area of dead cells also increased significantly. Perhaps because when the amount of PLLA content is too high, its degraded metabolites can affect the normal growth state of cells. Therefore, the PCL/PLLA strapping bands with 20% addition of PLLA showed the best proliferative capacity of rBMSCs.

**Fig. 4:**
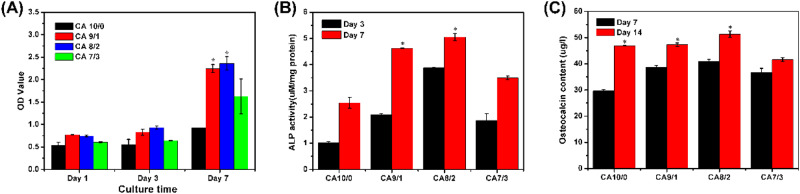
**A** OD value of rBMSCs on the strapping bands, **B** alkaline phosphatase activity of rBMSCs on the strapping bands, and **C** osteocalcin secretion expression of rBMSCs on the strapping bands (*n* = 5, *p < 0.05)

The absorbance (OD) value is used as an indicator of cell proliferation. The larger the OD value, the better the survival of the cells on the strapping, the lower the toxicity, and the smaller the effect on cell proliferation. As shown in Fig. 4A, the OD value of the four groups of strapping bands increased with the culture time. the OD value of the four groups did not significantly increase from day 1 to day 3. By the seventh day, the OD value of the CA9/1 and CA8/2 strapping bands has increased a lot, and CA8/2 strapping bands was better. The OD value of CA7/3 strapping bands is consistent with the result of Fig. 3, which showed weak proliferation capacity due to excessive PLLA content. This suggests that PCL/PLLA strapping bands has little toxicity to rBMSCs and can promote the proliferation of rBMSCs.

Alkaline phosphatase (ALP) activity is another important indicator in evaluating the capacity of rBMSCs. It is an enzyme protein secreted by osteoblasts and is one of the early markers of osteoblast differentiation [30, 31]. The ALP activity of the rBMSCs on the PCL/PLLA strapping bands with different mass ratios is shown in Fig. 4B. ALP activity in the four strapping bands increased with time, and the differences between groups were consistent with the results of Figs. 3 and 4A, with CA8/2 strapping bands having the largest ALP activity and potential osteogenic differentiation capacity.

The osteocalcin (OC) secretion expression of the rBMSCs on the four groups of strapping bands was tested through ELISA. OC is synthesized and secreted by osteoblasts, can maintain normal bone mineralization, and protect the cartilage. OC recombinant protein detection can directly reflect the condition of osteoblasts activity and bone formation, its high expression is one of the marks of osteoblasts into the functional phase [32, 33]. Figure 4C shows OC content secreted by cells that cultured on strapping bands with different mass ratios on days 7 and 14. With the increase of cell culture time, OC secretion also increased, and rBMSCs cultured on CA8/2 strapping bands showed higher OC secretion on days 7 and 14 than that on other groups, and showed better osteogenic differentiation ability.

### In vitro degradation test of PCL/PLLA strapping bands

**Fig. 5:**
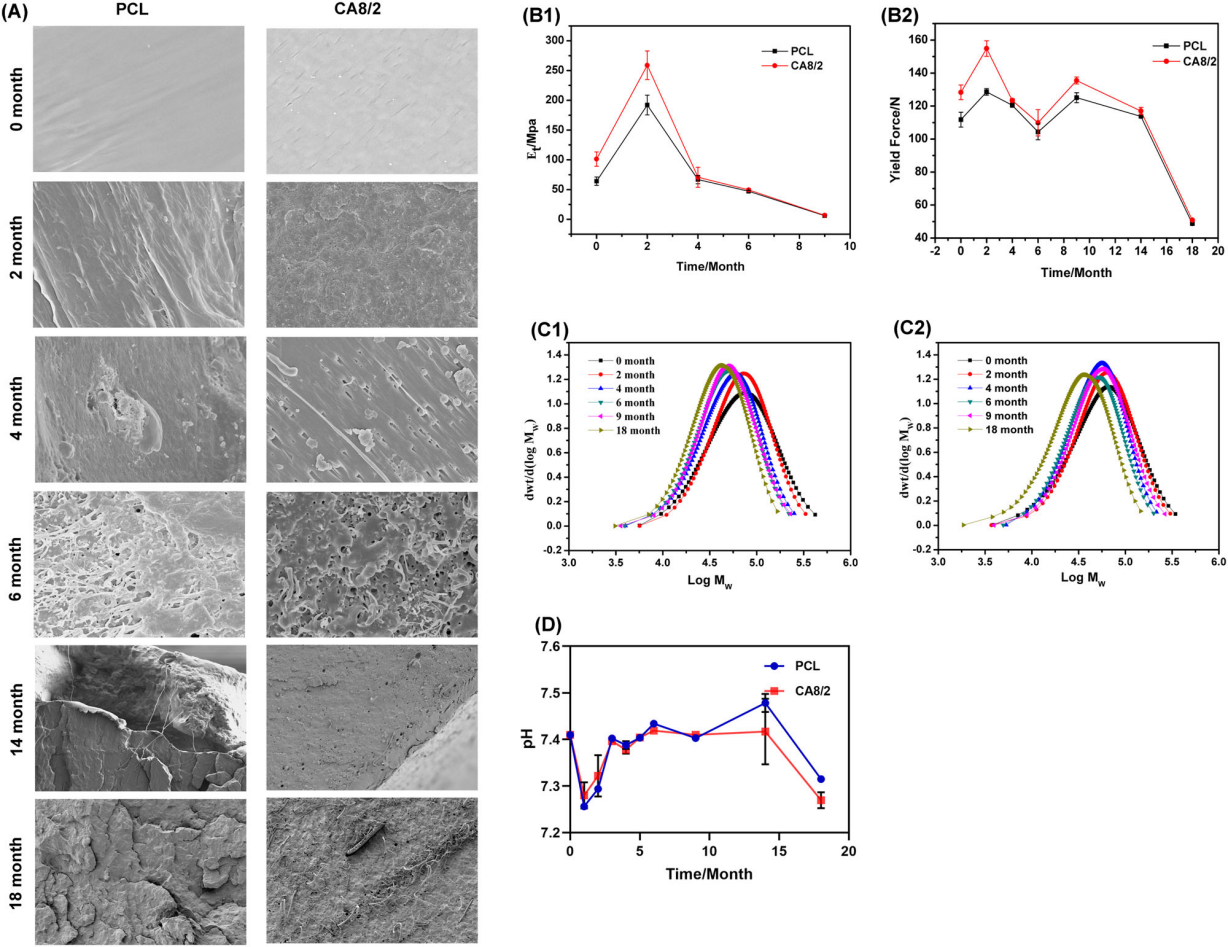
**A** SEM image for band binding during degradation (10 μm), **B1** Young’s modulus change curve during strapping bands degradation, **B2** yield force change curve during degradation, **C1** gel permeation chromatography for PCL strapping bands during degradation, **C2** gel permeation chromatography for CA8/2 strapping bands during degradation, and **D** pH change curve for band degradation in 37 °C PBS

The morphology of the strapping bands at 0, 2, 4, 6, 14, and 18 months in vitro is shown in Fig. 5A, and the undegraded PCL and CA8/2 sections are flat. The degradation of the PCL strapping bands showed a small number of unobvious cracks for 2 months, and the CA8/2 strapping bands showed a more obvious sea-island structure and some small pores due to the initial degradation of PLLA [34]. Significant cracks and pore-like structures were seen in the PCL and CA8/2 strapping bands sections for 4 months, with larger cracks and pores in the CA8/2 strapping bands sections, and a small number of spheres appeared, which may be small lactate molecules formed by degradation. The PCL strapping bands section of degradation for 6 months displays pore structures of different sizes, and the CA8/2 strapping bands section can see large pores and filament material at the 2-phase demarcation [35]. The surface of the PCL strapping bands band that has been degraded for 14 months has very obvious large cracks, with very fine filamentous material between the cracks, and still large pores on the surface of the CA8/2 strapping bands band, and spherical and fibrous material between the pores. More cracks are visible on the surface of the PCL strapping bands, and the CA8/2 strapping bands’ surface is covered with dense pores and disordered fibrous after 18 months of degradation.

The change curve of Young’s modulus and yield force of PCL and CA8/2 strapping bands during degradation is shown in Fig. 5B1, B2. The Young’s modulus and yield force of CA8/2 are higher than PCL before degradation, indicating that the addition of PLLA enhances the stiffness of PCL. After 2 months of degradation, the Young’s modulus and yield force of the two groups of strapping bands were significantly enhanced. It may be that the precipitation of the degradation product at the buckle made the sawtooth inside the buckle fit from the initial gap, so the reverse increase phenomenon after degradation appeared. After 4 months of degradation, the Young’s modulus of the two strapping bands was the same, and then the decline rate was consistent. After the 9th month, the strapping bands could not test the Young’s modulus. The decrease in tensile modulus can effectively avoid stress shielding and facilitate new bone growth after the 4th month. The yield force of the two groups also increased slightly in the ninth month after the degradation, which may be that the accelerated degradation at this time produced a large number of oligomers, and fibrous material gathered at the lock, and the serrated reached the surplus fit and this phenomenon appeared.

Relative to mass loss and crystallinity change, the molecular mass is the most accurate indicator for monitoring PCL hydrolysis [36]. The GPC map of 0, 2, 4, 6, 9, and 18 months of PCL and CA8/2 strapping bands are shown in Fig. 5C1, C2, the peak of all GPC curves shift to the left, which indicates that the weight-average molecular weight of the strapping bands has been decreasing. After 18 months of degradation, the weight-average molecular weight of the PCL strapping bands decreased from 65963 to 48568 Da, by about 26.4%, and the weight-average molecular weight of the CA8/2 strapping bands decreased from 79778 to 40235 Da, by about 49.6%. The weight-average molecular weight decay of CA8/2 strapping bands is nearly half faster than PCL strapping bands, indicating that the addition of PLLA significantly accelerated the rate of PCL degradation.

As shown in Fig. 5D, using the PCL strapping bands as a control group, we investigated the pH value of the CA8/2 strapping bands solution. The normal value of human pH should fluctuate between 7.35 and 7.45. According to the pH-value change curve, within the first two months, the pH values of both PCL and CA8/2 strapping bands decreased below 7.35. It is possible that the degraded PLLA produces small lactate molecules, which make the pH values below the normal range of the human body [37, 38]; From 3–14 months, the pH value of CA8/2 was between 7.35 and 7.45. The pH value of PCL strapping bands was slightly higher than 7.45 at the 14th month; From 14 to 18 months, the pH values of the solution of PCL and CA8/2 strapping bands reached 7.33 and 7.26, respectively, It is possible that the dissolution of some acidic oligomers produced by PCL degradation in PBS makes the pH value lower than the normal range of the human body [39, 40]. It can also be found that all the CA8/2 strapping bands were lower than the pH value of the PCL strapping bands from the 4th month, because the added PLLA produced more acidic oligomers. However, the small molecule products produced by the PCL and PLLA in vivo degradation can be absorbed and excreted [24], the further degradation in vivo is needed.

### In vivo safety assessment of PCL/PLLA strapping bands

The blood collection analysis of the beagle half month after operation as shown in Table 1, from biochemical blood routine test index, postoperative half month beagle dog without inflammation, the inflammatory index in normal beagle biological reference interval, super sensitive C protein also proved no postoperative beagle infection, associated with bone procalcitonin, total 25-hydroxy vitamin D, osteocalcin and collagen degradation products are also in the normal range. Therefore, there was no inflammatory reaction or infection after the implantation into the beagle dog.

**Table 1:** Postoperative blood test report for the beagle

Project/Unit	Result	Biological reference interval
White blood cell count/(10^9^/L)	8.55	4.90–14.80
Neutral cell count/(10^9^/L)	1.03	4.00–12.60
Lymphocyte count/(10^9^/L)	7.27	0.80–5.10
Monocyte count/(10^9^/L)	0.25	0.20–1.70
Monocyte ratio/(%)	2.90	1.3–4.8
Eosinophil ratio/(%)	0.00	0.0–0.3
Basophils ratio/(%)	0.00	0.0–1.0
Red blood count/(10^12^/L)	7.57	5.5–8.5
Hemoglobin/(g/L)	163	110–190
Hematocrit/(L/L)	0.53	0.35–0.6
Mean corpuscular volume/(fL)	69.4	60.0–70.0
Mean hemoglobin content/(pg)	21.5	21.1–25.3
Mean hemoglobin concentration/(g/L)	310	311–352
Red blood cell distribution widthSD/(fL)	37.3	34.0–56.0
Blood platelet count/(10^9/L)	222	117–460
Thrombocytocrit/(%)	0.30	0.01–0.40
Mean platelet volume/(fL)	13.5	9.4–18.5
Platelet distribution width/(fL)	19.2	9.0–21.0
High sensitivity C-reactive protein/(mg/L)	<0.5	cardiovascular disease<2; infection<10
Procalcitonin/(ng/mL)	<0.04	0–0.5
Total 25-hydroxyvitamin D/(ng/mL)	32.3	>30.0
Osteocalcin (N-M1D)/(ng/mL)	11.38	24–70
Collagen degradation product (β-CTX)/(ng/mL)	0.35	≤0.58

**Fig. 6:**
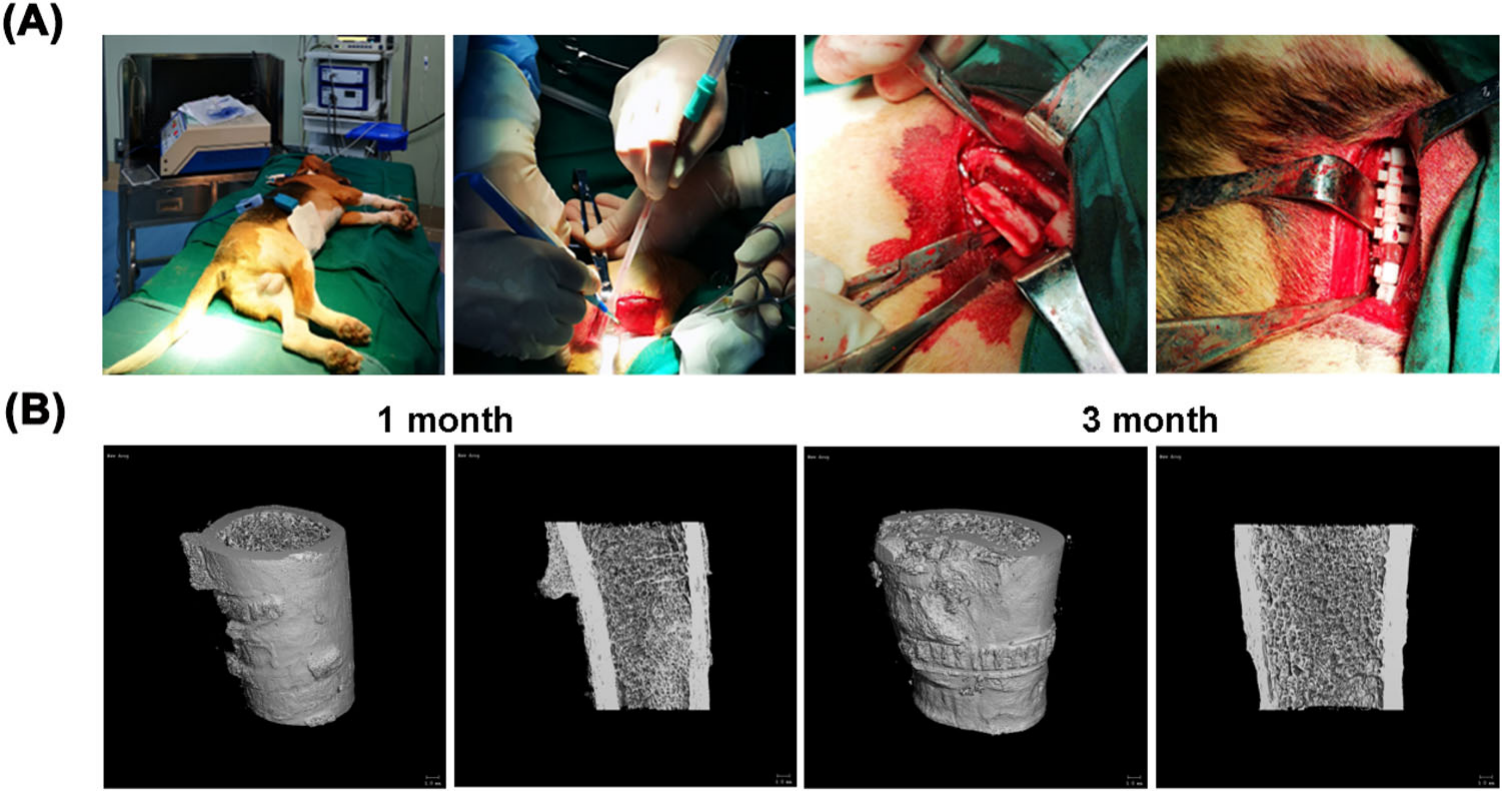
**A** The implantation process of strapping bands, **B** Micro-CT images of the beagle’s fracture recovery after the operation

Micro-CT images of the postoperative fracture healing in Beagle dogs are shown in Fig. 6. One month after the operation, it was found that the fracture line was basically invisible at the fracture site, and several callus appeared at the strapping bands site, which was a normal phenomenon in the fracture recovery period. The callus was still visible 3 months after the operation, but it was already very small. The bone surface still has serrated marks on the inner surface of the strapping bands, indicating that the PCL/PLLA strapping bands can still maintain considerable mechanical strength.

**Fig. 7:**
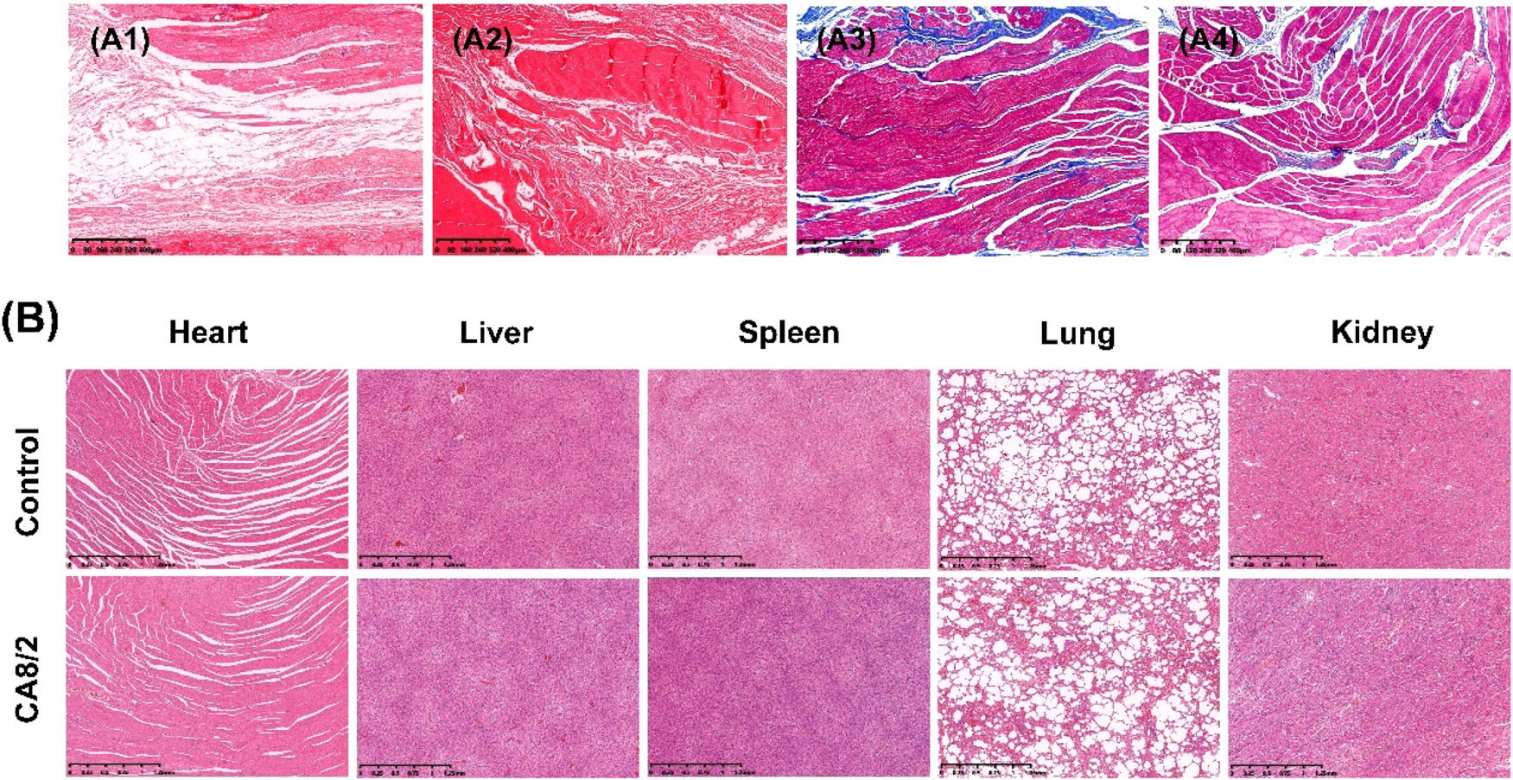
**A1** HE staining of peripheral connective tissue around fracture in external fixation group, **A2** HE staining of peripheral connective tissue around fracture in CA8/2 group, **A3** Masson staining of peripheral connective tissue around fracture in external fixation group, **A4** Masson staining of peripheral connective tissue around fracture in CA8/2 group (400 μm), **B** HE staining of main organs of the beagles in external fixation group and CA8/2 group (1.25 mm)

We collected the connective tissue around the strapping bands. The connective tissue was fixed, embedded, and prepared into sections, and then the sections were stained with HE and Masson. As shown in Fig. 7, there was no abnormal inflammatory reaction in CA8/2 group compared with the normal connective tissue. In order to further characterize the biological morphology of the connective tissue, the collagen fibers (blue) and the muscle fibers (red) of the connective tissue at the fracture site were characterized by Masson staining. Masson staining results of the control and experimental group of experiment group, no inflammation and hyperplasia, combined with HE staining results of dog organs and peripheral connective tissue, shows that the toxicity of PCL/PLLA strapping bands implantation is very low, no inflammation and hyperplasia, further proved the biosafety of PCL/PLLA strapping bands.

Figure 7B is the HE staining of main organs of the beagles (blue nucleus and red cytoplasm). The tissue morphology and density of the heart, liver, spleen, lung, and kidney of CA8/2 were after implantation compared with the external fixation group, and no abnormal inflammatory reaction or damage was found. Thus, PCL/PLLA strapping bands does not result in post-implantation in vivo toxicity.

## Conclusions

In this study, we prepared PCL/PLLA strapping bands with different mass ratios by injection molding, and tested the strength of different PCL/PLLA strapping bands. In vitro degradation experiment, we tested the micromorphology, mechanical properties, weight-average molecular weight, and pH value of PCL/PLLA strapping bands at various time points. The results indicated the addition of PLLA could maintain the pH value of the solution in the normal range of human body and accelerate the degradation speed of the strapping bands. In vitro cell experiments, we tested the proliferative capacity, alkaline phosphatase activity, and osteocalcin secretion expression of rBMSCs on PCL/PLLA strapping bands. The results proved that PCL/PLLA strapping bands adding 20% PLLA was nontoxic to rBMSCs and could promote osteogenic differentiation ability of rBMSCs. In vivo animal experiments further proved the biosafety performance of PCL/PLLA strapping bands. PCL/PLLA strapping bands had good therapeutic effect. It could maintain the original shape and volume and sufficient mechanical strength during the whole healing process of fracture. In this study, the medical grade PCL was modified as the base body by adding PLLA, which is expected to be used for the clinical treatment.
